# 3D Laser Nanoprinting of Optically Functionalized Structures with Effective-Refractive-Index Tailorable TiO_2_ Nanoparticle-Doped Photoresin

**DOI:** 10.3390/nano12010055

**Published:** 2021-12-25

**Authors:** Shichao Song, Yijie Li, Zhuofan Yao, Jie Li, Xiangping Li, Yaoyu Cao

**Affiliations:** Guangdong Provincial Key Laboratory of Optical Fiber Sensing and Communications, Institute of Photonics Technology, Jinan University, Guangzhou 511443, China; songsc@jnu.edu.cn (S.S.); liyijie@stu2018.jnu.edu.cn (Y.L.); yaozhuofan@stu2019.jnu.edu.cn (Z.Y.); tjieli@jnu.edu.cn (J.L.); xiangpingli@jnu.edu.cn (X.L.)

**Keywords:** titanium dioxide-based nanocomposite, optically functionalized nanostructures, laser nanoprinting, femtosecond laser, effective refractive index

## Abstract

The advanced direct laser printing of functional devices with tunable effective index is a key research topic in numerous emerging fields, especially in micro-/nano-optics, nanophotonics, and electronics. Photosensitized nanocomposites, consisting of high-index materials (e.g., titanium dioxide, TiO_2_) embedded in polymer matrix, are emerging as attractive platforms for advanced additive manufacturing. Unfortunately, in the currently applied techniques, the preparation of optically functionalized structures based on these photosensitized nanocomposites is still hampered by many issues like hydrolysis reaction, high-temperature calcinations, and, especially, the complexity of experimental procedures. In this study, we demonstrate a feasible strategy for fabricating micro-/nanostructures with a flexibly manipulated effective refractive index by incorporating TiO_2_ nanoparticles in the matrix of acrylate resin, i.e., TiO_2_-based photosensitized nanocomposites. It was found that the effective refractive index of nanocomposite can be easily tuned by altering the concentration of titanium dioxide nanoparticles in the monomer matrix. For TiO_2_ nanoparticle concentrations up to 30 wt%, the refractive index can be increased over 11.3% (i.e., altering from 1.50 of pure monomer to 1.67 at 532 nm). Based on such a photosensitized nanocomposite, the grating structures defined by femtosecond laser nanoprinting can offer vivid colors, ranging from crimson to magenta, as observed in the dark-field images. The minimum printing width and printing resolution are estimated at around 70 nm and 225 nm, indicating that the proposed strategy may pave the way for the production of versatile, scalable, and functionalized opto-devices with controllable refractive indices.

## 1. Introduction

As one of the most common strategies used in additive manufacturing, direct-laser printing (DLP), which employs the two-photon polymerization effect, has emerged as a promising technique for fabricating spatially resolved two-dimensional (2D) or three-dimensional (3D) structures [1,2,3,4,5]. Featuring sub-diffraction limit resolution, DLP can offer reliable light–matter interaction at the polymerization volume [6,7,8,9]. Relaying on the proper choice of photosensitized resins, various specified, multidisciplinary devices have been successfully fabricated and have evidenced their ability to deliver pre-designed functionalities [10,11,12,13,14], such as 3D photonics crystals [15,16,17], metallic structures [18,19,20], conductive devices [21,22], remote-responsive mechanical devices [23], etc.

Functional photosensitized nanocomposites incorporating large-index titanium dioxide (TiO_2_) in photoresin matrix are regarded as beneficial material candidates for the fabrication of micro-/nano-architectures with spatially varied optical response (e.g., scattering amplitude, phase, and polarization) in the visible and near-infrared range [16,24]. General processes for the fabrication of TiO_2_-based functional nanocomposites involve deposition and etching, which limits their application, mainly in fabricating 2D structures. Currently, in most common strategies reported for direct laser printing, TiO_2_-based nanostructures are demonstrated by employing liquid TiO_2_ precursors and acrylic titanium alkoxide [15,25,26,27,28,29], both of which demand post-heat-treatment [30] for forming inorganic amorphous TiO_2_ from the decomposition of organic precursors. Although an effective refractive index of TiO_2_-based nanocomposite can be obtained as high as 2.3, close to the value of crystal phase [15], the post-heat-treatment may cause undesired structural shrinking [30] and poor adhesion between the sample and the substrate, which may lead to unwanted optical functionalities. In addition, tunable refractive indices can be also be produced in nanocomposites by controlling the percentage of C=C bonds consumed during the polymerization process; therefore, they could be used to produce aberration-free and plane optical surface components [31]. However, the tuning range of the refractive index still needs to be improved beyond the order of 0.01 in this method.

In this study, we demonstrate a feasible approach for fabricating micro-/nanostructures with flexibly manipulated effective refractive indices by incorporating TiO_2_ nanoparticles in a matrix of acrylate resin, i.e., tailoring the TiO_2_ concentration of TiO_2_-based photosensitized nanocomposites. Through the implementation of this effective-refractive-index tailorable photosensitized nanocomposite, the direct printing of 3D optical nanoarchitectures can be achieved in a single attempt. For a nanocomposite with a TiO_2_ concentration up to 30 wt%, the refractive index can be as high as 11.3% (i.e., increasing from 1.50 of pure monomer to 1.67 at 532 nm). As a result, the optical response of the as-fabricated micro-/nanostructures could be tuned by directly changing the TiO_2_ concentration in the monomer matrix.

## 2. Materials and Methods

### 2.1. Synthesis of the Titanium Dioxide-Based Photosensitized Nanocomposites

A 3-methacryloxy propyl trimethoxy silane (MAPTMS, purchased from TCI Chemicals, Shanghai, China) is hydrolyzed in ethanol/hydrochloric acid solution (0.05 mol/L) with the weight ratio of 9:1 at 60 °C for 1 h with vigorous stirring. Next, the hydrolytic MAPTMS is added dropwise into ethanol suspension containing ~5 nm TiO_2_ nanocrystals or nanoparticles (purchased from Shanghai Aladdin Biochemical Technology Co., Ltd., Shanghai, China) with vigorous stirring for 6 h. Subsequently, the TiO_2_ nanoparticle sample is washed twice with ethanol to obtain the final surface-modified anatase TiO_2_ nanoparticle.

### 2.2. Synthesis of the Photosensitized Nanocomposite Photoresin

The MAPTMS-modified TiO_2_ nanoparticles are homogeneously mingled with photosensitized resin, which was composed of pentaerythritol tetraacrylate (PETTA, purchased from Sigma-Aldrich, Shanghai, China) as monomer and 0.5 wt% 7-diethylamino-3-thenoylcoumarin (DETC, purchased from Alfa Chemistry, Ronkonkoma, NY, USA) as photoinitiator.

### 2.3. 3D Laser Nanoprinting

The direct laser printing system is employed to produce the transdimensional structures from the TiO_2_-based photosensitized nanocomposite. A 1064 nm-femtosecond laser beam, originating in the 800 nm-wavelength Ti: Sapphire laser (repetition rate of 80 MHz, pulsewidth of 140 fs, Chameleon Ultra II, Coherent, Inc., Santa Clara, CA, USA) and optical parametric oscillators (OPO) (pulse width of 210 fs, Chameleon Compact OPO, Coherent, Inc., Santa Clara, CA, USA), is used to generate the 532 nm-femtosecond laser beam with a repetition of 80 MHz and a pulse width of 210 fs through the second harmonic generator (Coherent, Inc., Santa Clara, CA, USA). The radiative 532 nm-femtosecond laser beam is directly focused onto the sample mounted on a piezo multi-axis stage (P-563.3CD, Physik Instrumente (PI) GmbH & Co. KG, Karlsruhe, Germany) through an oil immersion objective lens (UPlanSApo, 100×/1.40 (Oil), Olympus, Shinjuku-ku, Japan). A CCD camera (MER-132-43U3M-L, Daheng, Beijing, China) that provides a bright field microscope magnified field of view in the visible light region is used to monitor the fabrication process of the structures.

The two-photon absorption occurs when a certain light intensity level is reached. The intensity *I* can be expressed as follows [32]:

(1) $$I=\frac{2P_{a}T}{R\pi w_{0}^{2}\tau }$$

where *P*_a_ is the average power, *T* is the objective transmission, *R* is the repetition, *w*_0_ is the radius at the beam waist, and *τ* is the pulsewidth. Herein, *T*, *R*, and *τ* are ~0.85 at 532 nm, 80 MHz, and 210 fs in our 532 nm-femtosecond laser printing system, respectively. Furthermore, the optical field under tight focus effect transforms from a circular to an elliptic shape due to the depolarization. Therefore, when the polarization of the incident beam is along the *y* direction, the *w*_x_ and *w*_y_ are 0.1721 × 532 nm and 0.2449 × 532 nm, respectively. Consequently, the intensity *I* at the focal spot is calculated with the average power before pupil as *I* = 2.84 × 10^6^·*P*_a_ (W/μm^2^).

### 2.4. Optical Properties of the Photosensitized Nanocomposites

Since the nanocomposite is composed of host matrix and embedded nanoparticles, its optical properties can be described by an effective-medium theory [33]. The most common effective-medium theory, the Clausius–Mossoti (CM) equation, is defined as:

(2) $$\varepsilon_{\mathrm{eff}}=\varepsilon_{\mathrm{resin}}\frac{1+\frac{16f}{d_{{\mathrm{TiO}}_{2}}^{3}}\alpha_{{\mathrm{TiO}}_{2}}}{1-\frac{8f}{d_{{\mathrm{TiO}}_{2}}^{3}}\alpha_{{\mathrm{TiO}}_{2}}}$$

where *ε*_eff_ is the effective permittivity of the nanocomposite, *ε*_resin_ is the permittivity of the host matrix, $\alpha_{{\mathrm{TiO}}_{2}}$ is the dipole polarizability of the TiO_2_ nanoparticles, $d_{{\mathrm{TiO}}_{2}}$ is the diameter of the sphere particle, *f* = 4/3·*N*·π ($d_{{\mathrm{TiO}}_{2}}$/2)^3^ is the volume fraction, and *N* is the number density of the TiO_2_ nanoparticles. This equation connects $\alpha_{{\mathrm{TiO}}_{2}}$ to the effective permittivity of the composite (*ε*_eff_). It is assumed that each particle can be described by a dipole moment $P_{{\mathrm{TiO}}_{2}}$ = $\alpha_{{\mathrm{TiO}}_{2}}$ ***E***_loc_, and that the local field (***E***_loc_) matches the external field (<***E***_loc_> = ***E***_ext_) only when the TiO_2_ nanoparticles are randomly distributed in an infinite host matrix [34,35]. However, the CM equation for the nanoparticles in a finite host matrix needs to be modified to express the polarizability $\alpha_{{\mathrm{TiO}}_{2}}$ in terms of the constituents’ properties, such as its size, shape, and refractive index. Thus, taking the Mie theory into account, the polarizability *α*_TiO_2__ can be described as [36]:

(3) $$\alpha_{{\mathrm{TiO}}_{2}}^{\mathrm{Mie}}=i\frac{3{\left(d_{{\mathrm{TiO}}_{2}}/2\right)}^{3}}{2x^{3}}a_{1}$$

where *a*_1_ is Mie coefficient of electric dipoles, and *x* is the size parameter (*x* = π·*n*_eff_·*d*/*λ*, where *λ* is the wavelength). By substituting Equation (3) into Equation (2), the Mie-modified Maxwell–Garnett (MG) theory can be applied. If the *a*_1_ is replaced by the expansion in Mie scattering theory, and all the first order terms are neglected, the polarizability can be simplified as in [33]:

(4) $$\alpha_{{\mathrm{TiO}}_{2}}={\left(d_{{\mathrm{TiO}}_{2}}/2\right)}^{3}\frac{\varepsilon_{{\mathrm{TiO}}_{2}}-\varepsilon_{\mathrm{resin}}}{\varepsilon_{{\mathrm{TiO}}_{2}}+2\varepsilon_{\mathrm{resin}}}$$

Next, the effective refractive index *n*_eff_ of this nanocomposite can be determined as:

(5) neff=εeff≈nresin(1+2πNRe(αTiO2))+i⋅nh2πNIm(αTiO2)

where *n*_resin_ is the refractive index of the host matrix. As a result, the scattering properties of this nanocomposite can be described by the Mie scattering theory, and the extinction (*σ*_ext_) and scattering (*σ*_scat_) cross-sections are:

(6) σextMie=λ22π∑n=0∞(2n+1)Re(an+bn)

(7) $$\sigma_{\mathrm{scat}}^{\mathrm{Mie}}=\frac{\lambda^{2}}{2\pi }\sum_{n=0}^{\infty }\left(2n+1)({\left|a_{n}\right|}^{2}+{\left|b_{n}\right|}^{2}\right)$$

where *a*_n_ and *b*_n_ are the electric and magnetic Mie coefficients, which correspond to different multipoles (*n* = 1: dipoles; *n* = 2: quadrupoles). Furthermore, these coefficients are functions of the effective refractive index. The absorption cross section (*σ*_abs_) follows from energy conservation as *σ*_abs_ = *σ*_ext_ − *σ*_scat_. For dilute solutions (single scattering limit), this makes it possible to directly determine the attenuation coefficient *γ*, since it can be directly obtained from the imaginary part of the effective refractive index.

(8) γMieext=4πλIm(neff)

### 2.5. Measurements of Dark-Field Forward Scattering Spectrum

A home-built forward scattering measuring setup is composed of a dark-field transmission microscope (Olympus BX53, Shinjuku-ku, Japan) and a spectrometer (Andor sR500i, Oxford Instruments, Belfast, UK) mounted on the extension port. The forward scattering spectra of the samples were collected through an objective lens (MPlanFL N, 5×/0.15, Olympus, Shinjuku-ku, Japan). The collection angle *θ* of the scattering field is related to the numerical aperture NA of the objective lens, which can be described as *θ* = 2 × arcsin(NA/*n*), where *n* is the refractive index between the objective lens and the sample.

## 3. Results and Discussion

**Figure 1:**
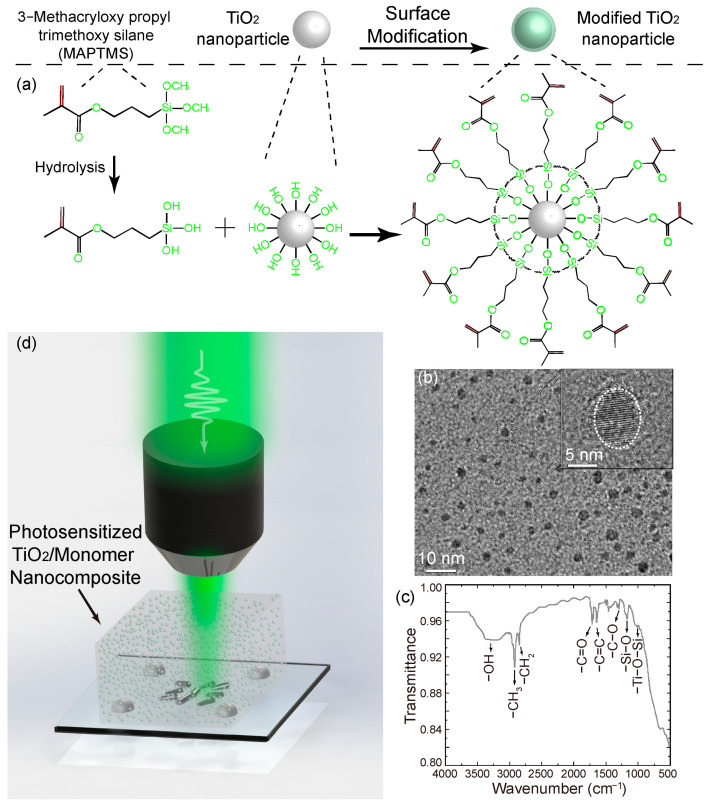
(**a**) Surface modification of TiO_2_ nanoparticles with MAPTMS. (**b**) The TEM images of modified TiO_2_ nanoparticles. (**c**) The FTIR spectrum of the modified TiO_2_ nanoparticles. (**d**) Schematic of the 3D femtosecond laser nanoprinting of TiO_2_-based photosensitized nanocomposites.

Due to its highly hydrophilic surface, the TiO_2_ nanoparticle cannot be well dispersed in organic mediums, such as dichloromethane, methyl methacrylate. In order to improve the dispersion, the surface-modified anatase TiO_2_ nanoparticles were obtained with the methods detailed in Section 2, as shown in Figure 1a. For the purpose of embedding the anatase TiO_2_ nanoparticle into the photoresin homogeneously to generate the photosensitized nanocomposite, the modified TiO_2_ nanoparticles/ethanol suspension was added into photosensitive resin with vigorous stirring for dispersion and ethanol evaporation, which imparted the photopolymerizable property to the TiO_2_ nanoparticles. The transmission electron microscope (TEM) image, presented in Figure 1b, shows that the size of most of the MAPTMS-modified TiO_2_ nanoparticles was around 5 nm. Moreover, a thin film, made of modified TiO_2_ nanoparticles, was formed by volatilizing dichloromethane from the modified TiO_2_ nanoparticle/dichloromethane suspension. Therefore, the spectrum captured by Fourier Transform Infrared (FTIR) Spectroscopy confirmed the successful grafting of MAPTMS organic groups on the surface of the modified TiO_2_ nanoparticles, presented in Figure 1c. Consequently, as shown in Figure 1d, the photosensitized resin synthesized with functional precursors was utilized for 3D femtosecond laser prototyping to achieve the manipulation of the effective refractive index by incorporating the TiO_2_-modified nanoparticles with different concentrations into the monomer matrix.

**Figure 2:**
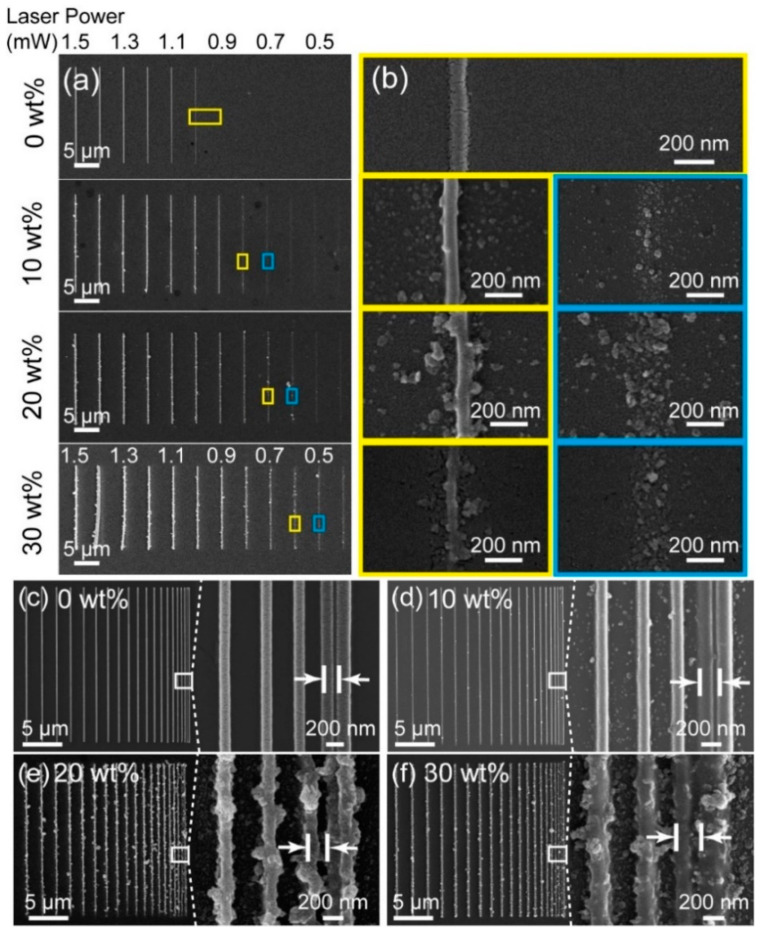
(**a**) The SEM image of the line structures at TiO_2_ nanoparticle concentration of 0–30 wt% with different printing power ranging from 1.5 to 0.4 mW, respectively. (**b**) The enlarged structures corresponding to the yellow and blue boxes in (**a**). (**c**–**f**) The images relevant to the printing resolution of TiO_2_-based structures with concentrations of (**c**) 0 wt%, (**d**) 10 wt%, (**e**) 20 wt%, and (**f**) 30 wt%. The space between the adjacent lines was designed to vary from 2 to 0.2 μm. The insets illustrate the zoomed-in results of the dashed-line areas.

The 532 nm femtosecond laser beam was directly focused onto the ~70 μm-thick TiO_2_-based photosensitized nanocomposite, which was sandwiched between the upper and the nether borosilicate cover slips (Fisherbrand). To further investigate the polymerization properties, a series of line structures were printed with power ranging from 1.5 to 0.4 mW, at a fixed scanning speed of 15 μm/s. Scanning electron microscopy (SEM) was employed to observe and analyze the morphologies of the printed structures. The threshold power was defined as the minimum power with which the line structure can be continuously printed via the direct laser writing or the printing technique. When the weight ratio was 0 wt%, i.e., the composite was merely the photoresin; as illustrated in Figure 2a, the polymerization only occurred at power above 1.0 mW, where the linewidth was ~103 nm. While the weight ratio varied from 10 wt% to 30 wt%, the printing threshold powers decreased from 0.8 to 0.6 mW (yellow boxes), so the single-line structures could simply be prepared with continuity. However, the predesigned line structures printed with lower powers were discrete structures consisting of isolated TiO_2_ particles (blue boxes), as confirmed in Figure 2b. Thus, the line widths were measured as 80, 76, and 70 nm with weight ratios of 10–30 wt%, respectively. Consequently, benefiting from excellent photoactive properties, TiO_2_ nanoparticles can serve as additional inorganic photoinitiators [37]. Therefore, with the joint contribution from these photoinitiators, the photopolymerized threshold power was reduced by 40% with the increase in the TiO_2_ concentration up to 30%. Furthermore, we elaborately designed and fabricated the nanostructures with the same line width and gradient spaces for revealing the printing resolution. Herein, printing resolution is defined as the left-edge-to-left-edge distance between two distinct adjacent lines, shown as the indicators. Furthermore, the space between the adjacent lines was designed to gradually decrease from 2 to 0.2 μm, and the rightmost gap was 0.2 μm. As presented in Figure 2c, the line width was ~156 nm at a power of 1.1 mW and the printing resolution was ~203 nm according to the indicator. When the concentration of TiO_2_ nanoparticles increased to 10 wt%, as shown in Figure 2d, the line width was ~155 nm with a power of 0.9 mW, and the resolution was ~230 nm, derived from the indicator in the enlarged SEM image. While the concentration reached 20 wt%, as shown in Figure 2e, the line width was ~153 nm at a power of 0.8 mW; however, the rightmost lines were almost indistinguishable; the resolution was measured as ~235 nm at the adjacent line structures on the left, marked as the indicator. When the concentration reached 30 wt%, as presented in Figure 2f, the line width remained at ~152 nm, at a power of 0.7 mW. Meanwhile, the rightmost lines were completely indistinguishable; therefore, the resolution was ~242 nm, according to the indicator in the image. As a consequence, the printing resolution could be still maintained at ~235 nm despite the increase in theTiO_2_ concentration.

**Figure 3:**
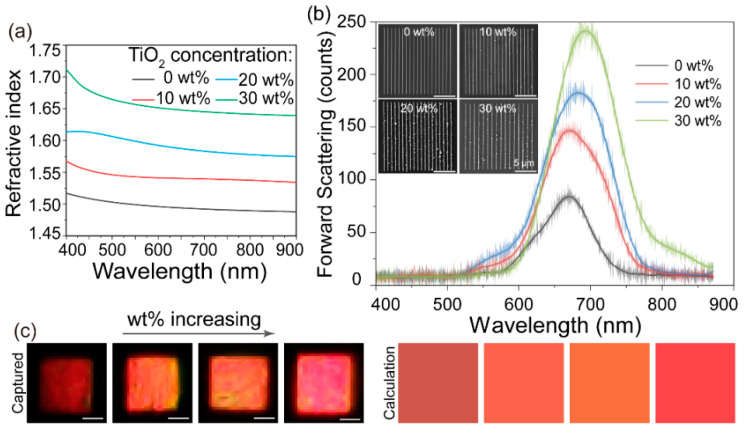
(**a**) The measured effective refractive index with four different concentrations via the ellipsometer. (**b**) The measured forward scattering spectra of four diffractive gratings with different concentrations. Insets: The SEM images of gratings with different TiO_2_ concentrations. (**c**) The calculated and measured dark-field color images captured by CCD camera. All the scale bars are 5 μm.

To investigate the optical properties of the TiO_2_-based nanocomposites, the effective refractive index *n*_eff_ was introduced to analyze the dispersion and optical response of the nanocomposites. The effective refractive index is closely related to the filling fraction of the TiO_2_ nanoparticles due to the Maxwell–Garnett formula in effective medium theory [34,35,38,39]; therefore, the optical properties of TiO_2_-based photosensitized nanocomposites can be tailored by altering the weight ratio of TiO_2_ nanoparticles. Here, we prepared four samples with different ratios of TiO_2_ nanoparticles, from 0 to 30%, by spin-coating on a silicon substrate and, subsequently, UV curing. The *n*_eff_ of the coated nanocomposites was experimentally characterized via the ellipsometer (M-2000, J. A. Woollam Co., Lincoln, NE, USA). As depicted in Figure 3a, the measured data, fitted using the Cauchy dispersion model, confirmed that *n*_eff_ can be controlled over a broad range by adjusting the weight ratio. When the concentration of TiO_2_ nanoparticles increased from 0% to 30%, *n*_eff_ increased over 11.3% (e.g., altering from 1.50 of pure monomer to 1.67 at 532 nm), which leads to spectra alterations.

In order to explore the spectral engineering of the as-printed nanostructures through the manipulation of *n*_eff_, four diffractive gratings were printed with the period of 1.15 μm and line widths of 152 nm@1.1 mW, 154 nm@0.9 mW, 155 nm@0.8 mW, and 153 nm@0.7 mW, respectively, as shown in the insets of Figure 3b. The dark-field forward scattering spectra with approximately the same criteria were characterized with the home-built forward scattering measuring setup. These samples were illuminated with a halogen white light source through an objective lens (×5, NA = 0.15). The diffractive angles of the as-printed diffractive gratings were around ±36.0° due to the diffractive equation, while the collecting angle of the objective lens was ±8.6°. Consequently, the spectra collected via the spectrometer were derived from the forward scattering of the jointed contribution of each line structure in the grating. As illustrated in Figure 3b, the collected forward scattering spectra (which can be described by the Mie scattering theory presented in Section 2) presented slight redshift and evident enhancement of scattering intensity with the gradual increase in the concentrations due to the increasing *n*_eff_ and effective optical path [33,35]. Moreover, the changes of the measured dark-field color images captured by the charge-coupled device (CCD) camera, shown in Figure 3c, confirm the alterations in the corresponding spectra. In addition, the calculated color images [40,41] with the measured structural and electromagnetic parameters were in good agreement with those of the experimental results. Hence, the optical activities of TiO_2_-based optical functional structures can be manipulated through tailored effective refractive indices and morphological structures.

**Figure 4:**
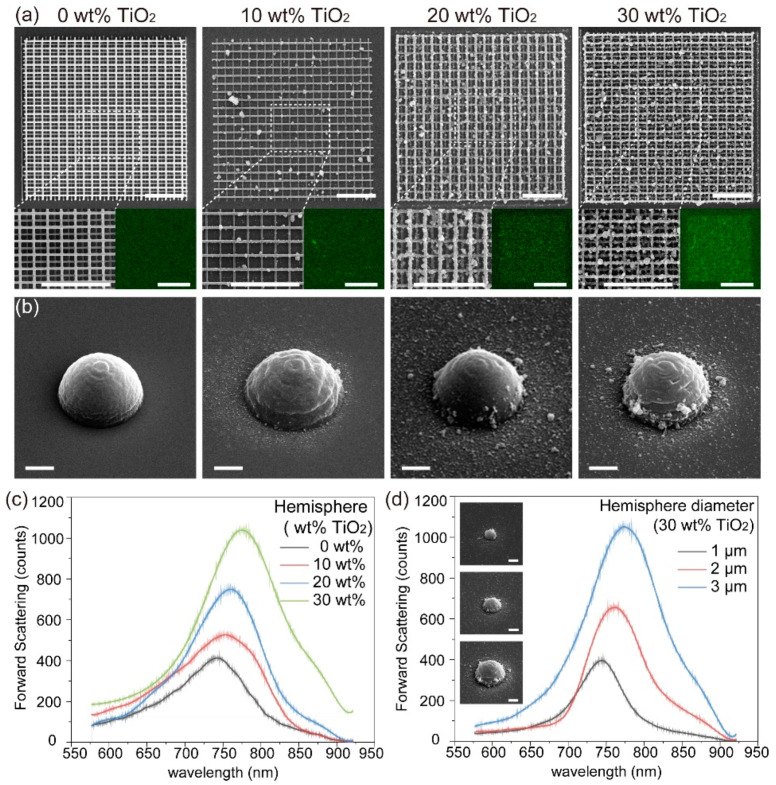
(**a**) The SEM images of the as-fabricated TiO_2_-based of woodpile-like structures. Insets: the enlarged SEM imaged of the dashed boxes and compositions of Ti element. The scale bars are 5 μm. (**b**) The SEM images of the as-fabricated hemispheres with expected diameters of 3 μm. (**c**) The corresponding forward scattering spectra of (**b**). (**d**) The forward scattering spectra of three different hemispheres with the same concentration, 30 wt%. Insets: SEM images of corresponding hemispheres. The scale bars in (**b**,**d**) are 1 μm.

Taking full advantage of the laser printing strategy, in addition, 3D functionalized structures were fabricated to manifest the capability of the two-photon polymerization of TiO_2_-based photosensitized nanocomposites. We conducted SEM and energy dispersive X-ray (EDX) analyses on the as-fabricated woodpile-like structures with the same period of 4 μm, as shown in Figure 4a. The line widths of these four woodpile-like structures were be confirmed in the zoomed-in images (insets of Figure 4a) as 156 nm@1.10 mW, 150 nm@0.89 mW, 170 nm@0.90 mW, and 162 nm@0.72 mW with the gradually increasing concentrations, respectively. Moreover, the EDX images illustrate the compositions of Ti element, which shows that the intensity increased as the concentrations of TiO_2_ nanoparticles increased. This confirms that the TiO_2_ nanoparticles were well embedded into the as-fabricated 3D structures. Furthermore, four hemisphere structures with different TiO_2_ concentrations were used to reveal the fabrication capacity and optical properties. As presented in Figure 4b, the expected diameters were set to be 3 μm, while the measured diameters were equal to 3.05 μm at 0 wt%, 3.10 μm at 10 wt%, 3.06 μm at 20 wt%, and 3.07 μm at 30 wt% with powers of 1.2, 1.0, 0.9, and 0.8 mW, respectively. The corresponding forward scattering spectra, illustrated in Figure 4c, indicate that the resonant wavelength redshifted and that the scattering intensity increased due to the increase in the effective optical path and *n*_eff_ [33,35]. Furthermore, three hemisphere structures with different diameters at the same concentration of 30 wt% were also fabricated to demonstrate the relationship between the scattering and the sizes, as depicted in Figure 4d. When the diameter varied from 1 to 3 μm, the resonant wavelength possessed a slight redshift due to the scaling effect of optical response and the scattering intensity increased with the enlargement of the structural size. The results further confirm the capability of 3D additive manufacturing with TiO_2_-based photosensitized nanocomposites, indicating its potential application in stereo-optical structures, such as spherical or aspherical lenses, 3D photonic crystals, etc.

## 4. Conclusions

In summary, we developed a flexible approach to generating TiO_2_-based optically functionalized nanostructures with tailorable refractive indices using the laser nanoprinting technique. Fully optical direct fabrications of TiO_2_-based 3D nano-architectures were achieved in a single attempt, which avoided the influence of the post-heat-treatment. The effective refractive index of the photosensitized resin could be gradually manipulated from 1.50 to 1.67 (an increment up to 11.3%) through the control of the TiO_2_ concentration in the photosensitized nanocomposites. The photopolymerized threshold power could be reduced from 1.0 mW to 0.6 mW, accompanied by a reduction in the line width from ~103 to ~70 nm. Therefore, the printing resolution increased to ~235 nm due to the near-field enhancement of the TiO_2_ nanoparticles. Hence, with the gradual increase in the TiO_2_ concentration, the scattering spectra of the as-printed 2D and 3D structures presented redshift and increased scattering intensity, which confirmed the feasibility of the spatially varying optical response of 3D laser-printed, optically functionalized structures based on TiO_2_-based photosensitized nanocomposites with tailored effective refractive indices. As a consequence, these nanocomposites could be promising candidates for producing low-threshold-polymerization-powered, large-area-miniaturized, compact, and integrated nanodevices in the fields of transdimensional optical manipulation, optical data storage, imaging, etc.

## Data Availability

The data presented in this study are available on request from the corresponding author.
